# Growth monitoring and promotion practices among health workers may be suboptimal despite high knowledge scores

**DOI:** 10.1186/s12913-019-4103-4

**Published:** 2019-04-29

**Authors:** Issahaku Sulley, Abdul-Razak Abizari, Zakari Ali, Wisdom Peprah, Hamshawu Gombilla Yakubu, Wilfred W. Forfoe, Mahama Saaka

**Affiliations:** 1 Department of Nutritional Sciences, School of Allied Health Sciences, University for Development Studies, P O Box 1883, Tamale, Ghana; 2 Impact Malaria Project, 14 Ollenu Street, East Legon, PMB 18, Accra, Ghana

**Keywords:** Growth monitoring, Child health records, Child welfare clinic, Growth chart, Knowledge and practices

## Abstract

**Background:**

The child health record booklet (CHRB) is a powerful tool for screening children under five and for education of caregivers by health workers. The objective of the present study was to assess the knowledge and utilization of CHRB by mothers and health workers in child growth monitoring and promotion (GMP) in the East Mamprusi Municipal, Northern region, Ghana.

**Methods:**

A descriptive cross-sectional study was conducted among mothers attending child welfare clinics (CWC) and health workers providing GMP at CWC. Observational checklists were used to assess 73 CHRB on the completeness and correctness of growth charts. Mothers and health workers’ knowledge on essential components of CHRB were assessed with a questionnaire.

**Results:**

Weight measurements were correctly recorded in all booklets analyzed. Even though a greater proportion (70.7%) of health workers exhibited high knowledge scores on the interpretation of the essential components of the CHRB,most of the charts analyzed were not completely filled (72.6%) but rather correctly filled (74.0%). Mean knowedge score (3.4 ± 1.3) on growth charting was low among mothers who attend GMP. Work overload (26.1%), inadequate supply of CHRB (26.1%) and vaccine shortages (18.7%) were concerns raised by health workers on the effective usage of the CHRB.

**Conclusion:**

Knowledge scores on the child health record booklets among health workers and mothers in this part of northern Ghana were high but charting of growth of children was sub-optimal among health workers.

**Electronic supplementary material:**

The online version of this article (10.1186/s12913-019-4103-4) contains supplementary material, which is available to authorized users.

## Background

Malnutrition constitutes a major public health concern worldwide and serves as an indicator for assessing overall health [1]. Recent estimates indicate that 165 million of all children under 5 years worldwide are stunted and a further 52 million are wasted with Africa and Asia recording the highest burden [2]. Whereas 14% of the global population is estimated to be undernourished, the prevalence of undernutrition is about 33% in sub-Saharan Africa [3].

In Ghana, reports on child under nutrition are not much different from the prevalence of sub-Saharan Africa. Among Ghanaian children under five years, 19% are stunted, 5% are wasted and 11% underweight [4]. In comparative view, Northern Ghana presents highest prevalence of under-five malnutrition as against the southern part [4].

The compounding effects of all forms of malnutrition are devastating to the individual, family, community and the nation as a whole. Nutrition plays a critical role in promoting and maintaining optimal health throughout the life cycle [5].

The child health record booklet (CHRB) is a forty-four paged document that combines essential health information on the growth monitoring of a child, immunization, vitamin A supplementation, deworming medicine and other illnesses [6] with the ultimate aim of having healthy thriving children with good nutritional status. A larger proportion of the CHRB is comprised of health messages on infant and young child feeding practices and family planning (48%), information about expanded programme on immunization makes up about 7%, the growth charts form about 2% of the CHRB, whilst child personal data and nurses progress notes make up the rest of the booklet. The CHRB assists in the screening of children at risk of malnutrition and provides a simple, practical, cost-effective and convenient method of monitoring a child’s health [6]. The CHRB also helps to improve health through vaccination compliance and early identification of growth patterns. It serves as a mobile health data bank and a source of useful reference for both caregivers at home and healthcare professionals during consultations [7].The CHRB has been used for a number of years in many countries to help track health risks, vaccinations and other preventive health measures performed [8]. The booklet is a document capturing necessary health information about children and their families. The content of the booklet has been found effective in assessing the overall health status of children worldwide [9].

Growth monitoring component of CHRB is a preventive strategy which when implemented properly helps in early detection of any growth faltering [10]. Growth monitoring can suggest an entry point to preventive and curative health care and serves as a constituent of programmes in line with significant reductions in malnutrition and mortality. Child growth monitoring and promotion (GMP) is key in caring for children. This involves measurement of growth in children, recording and interpreting in order to provide counselling, therapy and follow up [10]. The booklet also presents a milestone for routine health management information system data, utilization and coverage of preventive health services over time [11].

Over the last two decades, the CHRB used in Ghana has undergone several changes in both content and presentation of the health messages; changes of name from “Road to Health Chart” to CHRB, inclusion of nutrition counseling, sex appropriate colour coded growth charts, parent’s pledge, structured communication and a certificate for rewarding mothers who regularly attend CWC. These changes seem to increase the volume of the booklet which can engage the time of health workers and may not lead to its full utilization.

Little is known of the utility level by mothers and health workers providing GMP in Ghana. Such data could be useful in improving growth monitoring and promotion in Ghana. The present study, therefore, aimed to assess the knowledge and utilization level of CHRB as a growth monitoring and promotion tool in northern Ghana.

## Methods

### Study area

The study was conducted in the East Mamprusi Municipal area of the Northern Region, Ghana. It is located in the north-eastern part of the region. The Municipal has a single rainfall between April and October every year with mean annual rainfall of 1000 mm to 1500 mm. The average annual temperature ranges from 27^0^ C to 35^0^ C depending on the season. More than 8 in ten (83.6%) of the employed population are into agriculture involving crop production, livestock and fish farming. The Municipal has a rural population of 81,850, representing 67.6%. Females represent 51% of the population and a proportion of 17.5% of the total population were children younger than five years [12]. The Municipal has 35 health facilities; 1 hospital, 4 health centres and 30 Community-based Health Planning and Services (CHPS) compounds that deliver child welfare clinic (CWC) services.

### Design, target population and sampling

A descriptive cross-sectional design was used for this study. The target population included health workers of the selected health facilities. They were included in this study if they were involved in child welfare clinic. Another target population was mothers with children younger than five years. Mothers were eligible for the study if they were attending child welfare clinic and their children possessed CHRB. In all, 73 mothers and 58 health workers were included in the study. The data were collected mainly from five health facilities on a day set for CWC for mothers with children below 5 years and health workers. Different days were set for health workers who were on outreach or not on duty during initial data collection in their facilities. The participating facilities were: Nagboo CHPS, Sakogu, Gambaga and Langbesi health centres, and Nalerigu hospital. The study has been described following STROBE guidelines (Additional file 1).

### Data collection tools and procedures

Data were collected on days set for CWC at the various facilities using two questionnaires and an observation check list. The questionnaires were developed purposely for this study and were pre-tested before final utilization. The first questionnaire targeted the health workers. It was divided into socio-demographic characteristics, knowledge level and challenges faced by health workers on the usage of the CHRB. The second questionnaire targeted mothers/caregivers with the following sections: socio-demographic characteristic, knowledge level of the caregiver/mother and what ought to be done to improve caregivers’ knowledge on the CHRB. Questions for the assessment of health workers and caregivers’ knowledge were designed based on GMP components of the 2010 revised CHRB.

A check list on the growth chart components of the CHRB was used to assess the completeness and correctness of the growth chart. The questionnaires for this study have been provided as an additional file (See Additional file 2).

### Measurement of knowledge of health workers and mothers

The knowledge level of health workers was measured on growth monitoring and promotion aspects of the booklet. They were asked to interpret the Z-score component of the child growth chart, interval of vitamin A immunization, normal birth weight and time for issuing CHRB to the mother. Mothers’ knowledge level was also measured on interpretation of the growth chart and the purpose of the CHRB.

### Assessment of completeness of the growth chart

Checklists were used to assess completeness of CHRBs. A chart was considered completely filled when these eight conditions were met: selection of correct chart for boys (blue) and girls (pink), date of birth written, birth weight written and plotted, subsequent weights written and plotted, weights plotted for completed months, joined current plots to previous plots, blank space for missed visits (no dots/no dashes) and use of pen for plotting and writing on chart.

### Data analysis

Data were captured and analyzed using statistical package of social sciences (SPSS) software version 21. Data are presented as frequencies and percentages for categorical variables and as means and standard deviations for the continuous variables. A total score was calculated for each health worker from the 13 knowledge-based questions. Each correct response was accorded 1 point else 0. Similarly, a knowledge score was generated for each mother on 7 knowledge-based questions. A health worker scoring above mean knowledge score was considered knowledgeable. A mother scoring above the mean score was also considered knowledgeable.

A growth chart was considered correctly filled when at least 75 % of the criteria for assessing completeness were met.

## Results

### Socio-demographic characteristics of health workers and mothers

The study involved 58 health workers with a mean age of 29.1 ± 4.0 years. Majority (87.9%) of them had attained tertiary education and acquired certificates (69.0%). Most (56.9%) of them were female and Christian (65.5%). Only 6.9% of the health workers had at least eight years working experience.

Seventy-three mothers participated in the study. The majority (59.0%) of mothers were aged between 20 and 30 years, practiced Islamic religion (71.2%) and were married (97.3%). The largest proportion (47.9%) of mothers was from the Mamprusi ethnic group and more than half of them had formal education (52.0%). More than six in ten of the mothers had 1 to 3 children, while only 19.0% had no engagement in income generating activities (Table 1).

**Table 1:** Socio- demographic characteristics of health workers and mothers

Characteristic	Frequency	Percentage
Characteristics of health workers		
Age		
20–30	44	75.9
31–40	13	22.4
> 40	1	1.7
Sex		
Female	33	56.9
Male	25	43.1
Religion		
Christianity	38	65.5
Islam	20	34.5
Education level		
SHS	7	12.1
Tertiary	51	87.9
Highest qualification		
Certificate	40	69.0
Diploma	18	31.0
Ethnicity		
Mamprusi	28	48.3
Akan	9	15.5
Frafra	8	13.8
Other	13	22.4
Designation		
Community health nurse	18	31.0
General nurse	13	22.4
Health assistant	17	29.3
Other	10	17.3
Working experience		
1–2 years	25	43.1
3–5 years	13	22.4
6–8 years	16	27.6
> 8 years	4	6.9
Characteristics of caregivers/mothers		
Age		
20–30	43	59.0
31–40	25	34.2
> 40	5	6.8
Marital status		
Married	71	97.3
Divorced	2	2.7
Religion		
Christianity	19	26.0
Islamic	52	71.2
ATR	2	2.8
Educational level		
No education	35	48.0
Primary	13	17.8
JHS	12	16.4
SHS	9	12.3
Tertiary	4	5.5
Ethnicity		
Mamprusi	35	47.9
Frafra	11	15.1
Moshie	7	9.6
Other	20	27.4
Children		
1–3	47	64.4
4–6	22	30.1
7–9	4	5.5
Occupation		
Agriculture	26	35.6
Trader/ vendor	17	23.3
Service workers	10	13.7
Healthcare worker	1	1.4
None	19	26.0

### Knowledge level of health workers on the CHRB

Health workers’ understanding of the components of the CHRB is fundamental for GMP activities. This study revealed a majority (63.8%) of respondents indicating that the CHRB was issued at delivery. Almost 97 % also knew a child with birth weight less than 2.5 kg needed special care. Unexpectedly, 13.8% of health workers did not know that the colour of the growth chart for girls and boys are pink and blue respectively. It was also found that less than 25% of the health workers did not know that a child whose weight remain stagnant or drops for two consecutive visits should be referred for further attention. More than two-thirds of the health workers exhibited knowledge on the interpretation of each of the five Z-score points on the growth chart (severe underweight, moderate underweight, normal weight, overweight and obesity). Based on the 13 knowledge-based questions used, the mean knowledge score was 10.2 ± 1.8 with more than seven in ten of them scoring above the mean (Table 2).

**Table 2:** Knowledge level of health workers on the CHRB

Items	Frequency	Percentage
When is the CHRB issued?		
At delivery	37	63.8
First post-natal contact	21	36.2
A child with birth weight less than 2.5 kg needs special care?		
Agree	56	96.6
Disagree	1	1.7
Not sure	1	1.7
A baby may usually be fully immunized at age two?		
Agree	48	82.8
Disagree	5	8.6
Not sure	5	8.6
Vitamin A administration is six months interval?		
Agree	51	88.0
Disagree	1	1.7
Not sure	6	10.3
Growth chart for girls and boys are pink and blue respectively?		
Agree	48	82.8
Disagree	8	13.8
Not sure	2	3.4
If child weight remains the same for more than two consecutive times what must be done?		
Refer	44	75.9
Counsel	14	24.1
Which of the directions of a curve in the growth chart requires immediate action?		
Flat	4	6.9
Up	1	1.7
Down	53	91.4
A child whose WAZ is below −2 lines on the chart is?		
Normal	3	5.2
Underweight	47	81.0
Wasted	8	13.8
A child whose WAZ is between −2 and + 2 on the chart is?		
Normal	41	70.7
Underweight	9)	15.5
Wasted	8	13.8
A child whose WAZ is below −3 lines on the chart?		
Underweight	15	25.9
Severe underweight	43	74.1
A child whose WAZ is above +2 but less than + 3 is?		
Normal	11	19.0
Underweight	9	15.5
Overweight	38	65.5
A child whose WAZ is above + 3 lines on the chart is?		
Normal	1	1.7
Obese	43	74.1
Overweight	14	24.2
For a child whose WAZ is above + 3 lines on the chart?		
He/she require immediate action	43	74.1
The child is growing	15	25.9
Knowledge score, mean ± SD	10.2 ± 1.8	
High Knowledge	41	70.7
Low knowledge	17	29.3

### Knowledge level of mothers on the CHRB

Seven knowledge-based questions were used to assess the knowledge level of mothers who attend CWC on the CHRB. Mothers mentioned the most common purposes of the CHRB to include use for immunization (18.1%), keep child health history (17.1%) and for school entry and birth certificate (19.4%) However, the knowledge on the purposes of monthly weighing and nutritional counseling were below expectation (12.8). Out of the seven variables assessed, a greater proportion (78.1) of mothers could mention at least 3 variables. Two-thirds of mothers perceived that their child weight was correctly plotted, with more than half of them (56.3%) backing their perception by tracing the child’s weight with the child’s age (Table 3).

**Table 3:** Knowledge level of mothers on the CHRB

Items	Frequency	Percentage
Knowledge on purposes of CHRB		
Monitor child’s growth and development	35	16.6
For school entry and birth certificate	41	19.4
For health history	36	17.1
For detection of growth faltering and malnutrition	37	16.1
Weighing and nutritional counselling	27	12.8
Immunization services	38	18.0
Plotted weight properly joined?		
Yes	27	56.3
No	14	29.2
Partially	7	14.5
Nature of the curve		
Rising	24	32.9
Falling	6	8.2
Flat	2	2.7
Rising and falling	5	6.9
Cannot interpret	36	49.3
Interpretation of the curve		
Correct	36	49.3
Incorrect	37	50.7
Mean ± SD knowledge score	3.4 ± 1.3	
High knowledge	57	78.1
Low knowledge	16	21.9
Perceived correctly plotted curve by mothers		
Correct	48	65.8
Incorrect	25	34.2
Perceived reasons for correctly plotted		
Plotted vertically against child age	20	41.6
Weight corresponds with child’s age	27	56.3
Don’t know	1	2.1

### Correctness and completeness of filling the growth chart

We assessed the completeness and correctness of the growth chart with a number of variables. All the growth charts analyzed were sex appropriate, with a little above 78 % of the charts having dates of birth written appropriately. A little above 45 % of charts had no birth weights written and plotted. While about 80 % of subsequent weights after birth were written and plotted and 89% in completed months, only 19.2% of the charts were not defaulted. A greater proportion (74.0%) of the charts were correctly filled but only a little above twenty –seven percent were completely filled (Table 4).

**Table 4:** The completeness and correctness of filling the growth chart

Items	Frequency	Percentage
A correct chart is used		
Yes	73	100
No	0	0.0
Date of birth written		
Yes	57	78.1
No	16	21.9
Birth weight written and plotted		
Yes	40	54.8
No	33	45.2
Subsequent weight written and plotted		
Yes	58	79.5
No	15	20.5
Completed month plotted		
Yes	65	89.0
No	8	11.0
Plot (dot) well joined		
Yes	49	67.1
No	24	32.9
Defaulting visit left blank		
Yes	54	74.0
No	5	6.8
No defaulter	14	19.2
Plotting done with pen		
Yes	73	100.0
No	0	0.0
Chart filled completely		
Yes	20	27.4
No	53	72.6
Chart filled correctly		
Yes	54	74.0
No	19	26.0

### Actions to improve caregiver knowledge on proper utilization of the CHRB and retention of the previous booklet

The majority of the caregivers (89.0%) opined that regular education on the interpretation of the growth chart could help improve their utilization. Mothers were of the view that health workers usually do not inform them about the performance/growth of their children after weighing sessions neither are they asked to interpret the growth chart.

Of all the caregivers recruited for the study, 82.2% of them still possessed their previous child’s health record booklet with majority of those previous booklets being well kept (71.7%) in clean coverings, an indication of the importance mothers attach to the CHRB (Table 5).

**Table 5:** Actions to improve mother’s knowledge on the usage of CHRB and retention of previous booklet

Items	Frequency	Percentage
Mothers educated on the growth chart by health workers?		
Yes	36	49.3
No	37	50.7
What can be done to improve mother’s knowledge on the CHRB?		
Education on interpretation of the curve	65	89.0
Training on plotting of the curve	7	9.6
Don’t know	1	1.4
Previous CHRB present?		
Yes	60	82.2
No	13	17.8
Condition of previous CHRB		
Well kept	43	71.7
Covered	9	15.0
Dirty	7	11.7
Oiled	1	1.6

### Challenges faced by health workers in the utilization of the CHRB and charting of the growth chart

Challenges in relation to the level of practical skills of the health workers in plotting and interpreting the growth chart were also identified in the present study. In particular, using the information appropriately to counsel mothers was a big challenge. A lot of mistakes in taking measurements and plotting on the growth chart and counselling the mothers were obvious.

Among the health workers interviewed, almost 23 % indicated lack of confidence or uncertainty on their accuracy as a factor for improper charting of child growth parameters on the WHO child growth chart. Close to twenty -one percent of health workers as well indicated that both wrong organization (pilling of booklets to be charted) and fear of supervisor’s embarrassment for making mistakes were also contributing factors to improper charting. It was also revealed that 19.1% of the health workers were of the view that fear of colleague’s embarrassment for making mistakes could also be a factor for impeding charting of the growth chart. Challenges with regards to proper usage of the CHRB were enormous, and ranged from shortage of booklets, work overload, through vaccines shortages as common frustrations voiced by health workers (Table 6).

**Table 6:** Challenges faced by health workers in the utilization of the CHRB and charting of the growth chart

Utilization of CHRB	Frequency^a^	Percentage
Work overload	35	26.1
Shortage of staff	22	16.4
Shortage of vaccines	25	18.7
Shortage of CHRB	35	26.1
Irregular capacity building	7	5.2
Caregiver default	10	7.5
Charting of the growth chart		
Lack of confidence	23	23.0
Lack of commitment	29	16.3
Wrong organization (pilling of chart to be done later)	37	20.8
Fear of colleague’s embarrassment for making mistake	34	19.1
Fear of supervisor embarrassment for making mistake	37	20.8

^a^Multiple responses were allowed

## Discussion

This study sought to assess the knowledge and utilization of growth monitoring and promotion among mothers and health workers in Northern Ghana. The main findings of the present study are that: most of the health workers exhibited high knowledge on the interpretation of the essential components of the CHRB but that did not translate into skills in filling the growth chart. Majority of the mothers were also knowledgeable on the essential components of the CHRB. While a greater proportion of the growth charts were correctly plotted, there was poor level of completeness of the growth chart among health workers. These results could indicate a threat to the GMP programme as correctness and completeness are essential for GMP to be effective in identifying cases that require attention [13]. GMP is one of the important activities undertaken by health workers, therefore, a sound knowledge of it is paramount to reducing the burden of malnutrition among children under five years. It is mostly the first point for the monitoring, screening and management of childhood illnesses [13]. Ashworth et al. [14] reported that correct recording and charting of a child’s data on the growth chart allows for proper comparison of the child’s growth to the reference and could ensure early detection of growth problems for appropriate action. This important role may be compromised as the current study revealed only a smaller proportion of the charts were completed.

The finding in this study relating to large proportion of inappropriate charting is not consistent with that of Brownlee [15] who reported appropriate charting and understanding of the growth chart by health workers. One would expect health workers who play a pivotal role in providing health information for mothers on the usage of the child health record booklet to be equipped with the necessary knowledge to impart and influence practice. Even though knowledge on the booklet was high and consistent with existing data from Ghana [16], unfortunately, translation into practice was problematic. A systematic review by Roberfroid et al. [17] reported nearly a third of health workers in developing countries having poor understanding of the growth chart. The high knowledge level of health workers in the present study may be due in part to periodic trainings provided by non-governmental organizations in the area. The Ghana Health Service also provides periodic workshops on GMP which could explain the knowledge level observed. Knowledge level of the health workers appeared to be high on vaccines and vaccine routines and were higher than those reported from South Africa [6] but were less prominent on growth indicators. This could be explained by the caliber of health workers engaged in GMP. The common providers of GMP in health outlets in Ghana are general nurses and community health nurses who might not have much training on growth indicators compared to other cadre of health workers such as registered nutritionists who normally do not operate at this level.

Mothers had good perceptions on the CHRB concerning the monthly routines. This perception may motivate caregivers to make regular attendance for GMP at the health facilities. Similar results are reported by Gyampoh [16] whose study in the Greater Accra region of Ghana showed mothers placed importance on having their children weighed monthly. In this current study, mother’s ability to interpret the growth charts was similar to the health workers educating them about the nature of the growth curve during weighing session as a standard guideline for GMP (49.3%). This is similar to a study in Belgium where health workers made efforts to educate caregivers on growth charts, which led to improvement of mothers’ comprehension of the chart despite lags in formal education [18]. This may mean increasing the time for interaction with caregivers during GMP could improve their knowledge on interpretation of the chart [19, 20]. Our finding highlights the importance of nutrition counseling in achieving the objectives of GMP. The success of GMP depends on the knowledge, dedication and cooperation of the mothers and health workers. However, health workers identified uncooperativeness among caregivers as a challenge to GMP activities and indicated mothers being in a hurry to attend to other responsibilities at the household. This challenge could be tackled by involving the partners/husbands and household heads in GMP activities to understand the benefits of the programme and hence allow their women more time for GMP. In addition, addressing issues of work overload [21] of health workers and logistics [22] could be important at improving GMP activities in northern Ghana.

This study considered the overall sample as a combination of health workers and mothers and not separate even though we have performed sub-group analysis. As majority of the health facilities engaged in CWC also render the service through outreach to neighbouring villages, it is common not to find most mothers at the facility level for CWC which resulted in the inclusion of few mothers in the study. Data collection at the facility level was necessary because we needed a well representation of health workers which could suitably be gotten at the facilities. As many mothers are involved in CWC than health workers, we find that our sample may well represent health workers in the Municipality than mothers/caregivers. Our data on caregivers could therefore, be limited to this sample but may be useful in understanding their perceptions and knowledge on the CHRB. In spite of these limitations, the present study has provided ample information on the GMP programme in the East Mamprusi Municipal.

## Conclusion

Knowledge scores on the child health record booklets among health workers and mothers in this part of northern Ghana were high but charting of growth of children was sub-optimal among health workers. There is a need for increased education of mothers on the CHRB and its correct usage among health workers engaged in GMP in northern Ghana.

## Additional files

Additional file 1: STROBE Checklist. Checklist of items that should be included in reports of cross-sectional studies. (DOCX 32 kb)

Additional file 2: Questionnaires. Questionnaires designed purposely for the study of growth monitoring and promotion among caregivers and health workers. (DOCX 30 kb)

## Abbreviations

CHPs: Community-based Health Planning Service
CHRB: Child Health Record Booklets
CWC: Child Welfare Clinic
GMP: Growth Monitoring and Promotion
SPRING: Strengthening Partnerships, Results and Innovation in Nutrition Globally
