# Supplementary material for: Growth monitoring and promotion practices among health workers may be suboptimal despite high knowledge scores
Source: BMC Health Serv Res. 2019 Apr 29;19:267. doi: 10.1186/s12913-019-4103-4 (PMC6489312; doi:10.1186/s12913-019-4103-4)
Supplement: Supplementary file 1 — STROBE Checklist. Checklist of items that should be included in reports of cross-sectional studies. (DOCX 32 kb) [file 12913_2019_4103_MOESM1_ESM.docx]

STROBE Statement—Checklist of items that should be included in reports of ***cross-sectional studies***

|  | Item No | Recommendation | Page No |
| --- | --- | --- | --- |
| **Title and abstract** | 1 | (*a*) Indicate the study’s design with a commonly used term in the title or the abstract | Page-2  Line-24 |
|  |  | (*b*) Provide in the abstract an informative and balanced summary of what was done and what was found | Page-2  Line-20 and line 36-38 |
| Introduction | | | |
| Background/rationale | 2 | Explain the scientific background and rationale for the investigation being reported | Page 3-5  Line 45-96 |
| Objectives | 3 | State specific objectives, including any prespecified hypotheses | Page-5  Line- 95-96 |
| Methods | | | |
| Study design | 4 | Present key elements of study design early in the paper | Page-6  Line 110-111 |
| Setting | 5 | Describe the setting, locations, and relevant dates, including periods of recruitment, exposure, follow-up, and data collection | Page-5  Line 99-109 |
| Participants | 6 | (*a*) Give the eligibility criteria, and the sources and methods of selection of participants | Page-6  Line 111-115 |
| Variables | 7 | Clearly define all outcomes, exposures, predictors, potential confounders, and effect modifiers. Give diagnostic criteria, if applicable | Page-7  Line 135-146 |
| Data sources/ measurement | 8* | For each variable of interest, give sources of data and details of methods of assessment (measurement). Describe comparability of assessment methods if there is more than one group | Page 7  Line 134-145 |
| Bias | 9 | Describe any efforts to address potential sources of bias | Page 14  Line 290-294 |
| Study size | 10 | Explain how the study size was arrived at | Page 6  Line 114-120 |
| Quantitative variables | 11 | Explain how quantitative variables were handled in the analyses. If applicable, describe which groupings were chosen and why | Page 7  Line 149-156 |
| Statistical methods | 12 | (*a*) Describe all statistical methods, including those used to control for confounding | Page 7-8  Line 147-156 |
|  |  | (*b*) Describe any methods used to examine subgroups and interactions | Page 7  line 135-146 |
|  |  | (*c*) Explain how missing data were addressed | N. A |
|  |  | (*d*) If applicable, describe analytical methods taking account of sampling strategy | N. A |
|  |  | (*e*) Describe any sensitivity analyses | N. A |
| Results | | | |
| Participants | 13* | (a) Report numbers of individuals at each stage of study—eg. numbers potentially eligible, examined for eligibility, confirmed eligible, included in the study, completing follow-up, and analysed | Page-8  Line 160 & 164 |
|  |  | (b) Give reasons for non-participation at each stage | N. A |
|  |  | (c) Consider use of a flow diagram | N. A |
| Descriptive data | 14* | (a) Give characteristics of study participants (eg. demographic, clinical, social) and information on exposures and potential confounders | Page-8  Line 160 -168 |
|  |  | (b) Indicate number of participants with missing data for each variable of interest | N. A |
| Outcome data | 15* | Report numbers of outcome events or summary measures | Page 8-9  Line 176-180  Page 9  Line 186-190  Page 9  Line 197-199  Page 11  Line 224-227 |
| Main results | 16 | (*a*) Give unadjusted estimates and, if applicable, confounder-adjusted estimates and their precision (eg, 95% confidence interval). Make clear which confounders were adjusted for and why they were included | N. A |
|  |  | (*b*) Report category boundaries when continuous variables were categorized | N. A |
|  |  | (*c*) If relevant, consider translating estimates of relative risk into absolute risk for a meaningful time period | N. A |
| Other analyses | 17 | Report other analyses done—eg analyses of subgroups and interactions, and sensitivity analyses | N. A |
| Discussion | | | |
| Key results | 18 | Summarise key results with reference to study objectives | Page 11  Line 232-238 |
| Limitations | 19 | Discuss limitations of the study, taking into account sources of potential bias or imprecision. Discuss both direction and magnitude of any potential bias | Page 14 Line 290-294 |
| Interpretation | 20 | Give a cautious overall interpretation of results considering objectives, limitations, multiplicity of analyses, results from similar studies, and other relevant evidence | Page 11-13  Line 235-283 |
| Generalisability | 21 | Discuss the generalisability (external validity) of the study results | Page 14  Line 292-297 |
| Other information | | | |
| Funding | 22 | Give the source of funding and the role of the funders for the present study and, if applicable, for the original study on which the present article is based | Page 16  Line 331-332 |

*Give information separately for exposed and unexposed groups.

**Note:** An Explanation and Elaboration article discusses each checklist item and gives methodological background and published examples of transparent reporting. The STROBE checklist is best used in conjunction with this article (freely available on the Web sites of PLoS Medicine at http://www.plosmedicine.org/, Annals of Internal Medicine at http://www.annals.org/, and Epidemiology at http://www.epidem.com/). Information on the STROBE Initiative is available at www.strobe-statement.org.
