# Supplementary material for: Growth monitoring and promotion practices among health workers may be suboptimal despite high knowledge scores
Source: BMC Health Serv Res. 2019 Apr 29;19:267. doi: 10.1186/s12913-019-4103-4 (PMC6489312; doi:10.1186/s12913-019-4103-4)
Supplement: Supplementary file 2 — Questionnaires. Questionnaires designed purposely for the study of growth monitoring and promotion among caregivers and health workers. (DOCX 30 kb) [file 12913_2019_4103_MOESM2_ESM.docx]

**QUESTIONNAIRE ON GROWTH MONITORING AND PROMOTION FOR CAREGIVERS/ MOTHERS**

**SECTION A: IDENTIFICATION**

Questionnaire ID: …………………………….

District………………………………………….

Sub-health district: ……………………………

Name of health facility: …………………………

Date of visit: ………………………………...

Serial number: ……………………………...

**SECTION B: SOCIO-DEMOGRAPHIC CHARACTERISTICS OF MOTHER/CAREGIVER**

1. How old are you? ………………. (years)
2. What is your religious background? (1) Christianity (2) Islam (3) ATR (4) Others, specify……………………
3. What is your marital status? (1) Single (2) Married (3) Divorced (4) Widowed (5) Separated
4. What is your ethnicity? (1) Dagomba (2) Gonja (3) Mamprusi 4) Akan (5) Other, specify………………………
5. What is your educational level (1) None (2) Primary (3) JHS (4) SHS (5) Tertiary?
6. What is your occupation (1) Agricultural work (2) Trader/vendor (3) Office worker (civil servant) (4) Service worker (Hairdresser, Seamstress etc) (5) Education/research (teacher) (6) Healthcare (Nurse etc) (7) Nothing (8) Other, specify?
7. How many children do you have? ..........................

**SECTION C: KNOWLEDGE LEVEL OF THE CAREGIVER/MOTHER ON THE UTILIZATION OF THE CHRB**

1. What is the purpose of the child health record booklet? ………………………………………………………………………………………………………………………………………………………………………………………………………………………………………………………………………………………………
2. Is your child’s weight correctly plotted on the chart? (1) Yes (2) No (**Note: if no skip to 12**)
3. Why do you think it is correctly plotted? ………………………………………………………………………………………………………………………………………………………………………………………………
4. Is the plotted weight properly joined? (1) Yes (2) No (3) Partially (**interviewer response)**
5. Were you informed about the nature of the curve during your visits to weighing sessions? (1) Yes (2) No
6. What is the nature of the curve? (1) Rising (2) Falling (3) Flat (4) Rising and falling

(5) Cannot interpret

1. Is the interpretation above correct? (1) Yes (2) No (**interviewer response)**
2. What can be done to improve your knowledge concerning the CHRB?

………………………………………………………………………………………………………………………………………………………………………………………………………………………………………………………………………………………………

1. Do you have your previous booklet? (1) Yes (2) No
2. What is the nature of the CHRB observed? (1) Well kept (2) Oiled (3) Dirty (4) Covered (To be completed by interviewer**)**

**QUESTIONNAIRE ON GROWTH MONITORING AND PROMOTION FOR HEALTH WORKERS**

**SECTION A: IDENTIFICATION**

Questionnaire ID: …………………………….

District………………………………………….

Sub-health district: ……………………………

Name of health facility: …………………………

Date of visit: ………………………………...

Serial number: ……………………………...

**SECTION B: SOCIO-DEMOGRAPHIC CHARACTERISTICS OF HEALTH WORKERS**

1. Sex: (1) Female (2) Male
2. Age: ……… (Years)
3. What is your religious background? (1) Christianity (2) Islam (3) ATR (4) Others, specify……………………
4. What is your ethnicity? (1) Dagomba (2) Gonja (3) Mamprusi (4) Akan (5) Other, specify………………………
5. What is your education level? (1) JHS (2) SHS (3) Tertiary
6. What is your professional qualification? (1) Certificate (2) Diploma (3) Degree (4) Others, specify …………………
7. Years of working experience: …………………. (years)
8. What is your professional designation? (1) Community health nurse (2) General nurse (3) Midwife (4) Heath assistant (5) Other, specify

**SECTION C: KNOWLEDGE LEVEL OF HEALTH WORKERS ON THE CHRB UTILIZATION**

1. When do you issue the child health record booklet? (1) At delivery (2) First post-natal contact (3) Other, specify …………….
2. A child with a birth weight less than 2.5 kg in the booklet means they need special care. (1) Agree 2) Disagree (3) Not sure
3. A baby may usually be fully immunized at age two. (1) Agree (2) Disagree (3) Not sure
4. Vitamin A administration is at six-month intervals. (1) Agree (2) Disagree (3) Not sure
5. In growth monitoring and promotion, the growth chart for boys and girls are blue and pink respectively. (1) Agree (2) Disagree (3) Not sure
6. In growth monitoring and promotion, if child weight remains the same for more than two consecutive times you should ;(1) Refer (2) Counsel (3) Not sure
7. Which of the following directions of curves in the growth chart requires immediate action? (1) (2) (3) ( 4)
8. How do you interpret a child whose weight- for- age z-score is below -2 line on the chart? (1) normal (2) Underweight (3) Wasted
9. How do you interpret a child whose weight- for- age z-score is below -3 line on the chart? (1) normal (2) Underweight (3) Severe underweight
10. How do you interpret a child whose weight- for- age z-score is between -2 line and +2 on the chart? (1) normal (2) Underweight (3) Wasted
11. How do you interpret a child whose weight- for- age z-score is above +2 line on the chart? (1) normal (2) Underweight (3) Overweight
12. How do you interpret a child whose weight- for- age z-score is above +3 line on the chart? (1) normal (2) Obese (3) Overweight
13. A child whose weight- for- age z-score is above +3 line on the chart? (1) Require immediate action (2) The child is growing well (3) The child is healthy

**SECTION D: CHALLENGES FACED BY HEALTH WORKERS IN THE UTILIZATION OF THE CHRB**

1. What are the challenges you go through in the proper utilization of the CHRB?
2. ………………………………………………………………………………….
3. ………………………………………………………………………………….
4. …………………………………………………………………………………..
5. ………………………………………………………………………………….
6. In using the growth chart, the following may be a reason for improper charting of the child’s weight. (Multiple response accepted­).
7. Lack of confidence (2) Lack of commitment (3) Wrong organization (pilling of cards do be done later) (4) Fear of colleagues embarrassment for making mistakes (5) Fear of supervisors embarrassment for making mistake (6)Others: …………………………………………………………..

**CHECKLIST FOR THE ASSESSMENT OF CORRECTNESS AND COMPLETENESS OF CHILD GROWTH CHART**

Please refer to child growth chart and record details of the growth curve as indicated below*.

| SNo | Item | Yes (1)  No (0) |
| --- | --- | --- |
| 1 | A correct chart is used (blue for boys and pink for girls) |  |
| 2 | Date of birth written? |  |
| 3 | Birth weight written and plotted? |  |
| 4 | Are the subsequent weights written and plotted? |  |
| 5 | Are there plots for completed months? |  |
| 6 | Are the plots well joined? |  |
| 7 | Are the default visits left blank? |  |
| 8 | Is the plotting done with pen? |  |

*To be completed by interviewer
